# Comprehensive Profiling of Cytokines and Growth Factors: Pathogenic Roles and Clinical Applications in Autoimmune Diseases

**DOI:** 10.3390/ijms26188921

**Published:** 2025-09-13

**Authors:** Anna Donniacuo, Arianna Mauro, Chiara Cardamone, Anna Basile, Paola Manzo, Jelena Dimitrov, Anna Lisa Cammarota, Liberato Marzullo, Massimo Triggiani, Maria Caterina Turco, Margot De Marco, Alessandra Rosati

**Affiliations:** 1 Cytokine Lab, Clinical Pathology Unit, Hospital Unit “Gaetano Fucito”, 84085 Mercato San Severino, Italyarosati@unisa.it (A.R.); 2 Department of Medicine, Surgery, and Dentistry, University of Salerno, Via Salvador Allende, 84081 Baronissi, Italy; ccardamone@unisa.it (C.C.); mtriggiani@unisa.it (M.T.); 3 Clinical Immunology and Rheumatology Unit, Department of Internal Medicine, University Hospital “San Giovanni di Dio e Ruggi d’Aragona”, University of Salerno, 84131 Salerno, Italy; 4 Department of Humanities, Philosophy and Education Sciences/DISUFF, University of Salerno, Via Giovanni Paolo II, 132, 84084 Fisciano, Italy

**Keywords:** cytokines, growth factors, autoimmune diseases

## Abstract

Autoimmune diseases are characterized by dysregulated adaptive immune responses leading to chronic inflammation and tissue damage. Cytokines and growth factors play central roles in modulating immune regulation, inflammation, and tissue repair, thereby representing critical biomarkers for the enhancement of diagnosis, prognosis, and therapeutic monitoring. This review provides a comprehensive overview of pro-inflammatory and anti-inflammatory cytokines, as well as growth factors, emphasizing their pathogenic roles and clinical relevance across various autoimmune diseases, including rheumatoid arthritis, psoriatic arthritis, ankylosing spondylitis, and connective tissue diseases such as systemic sclerosis, Sjögren’s syndrome, and systemic lupus erythematosus. Key pro-inflammatory cytokines—such as TNF-α, IL-1β, IL-6, IL-17, and IFN-γ—are examined regarding their contributions to disease progression and activity, alongside anti-inflammatory cytokines like IL-10 and IL-4, which regulate immune tolerance and inflammation resolution. Growth factors, such as TGF-β, are analyzed for their dual roles in immune modulation, fibrosis, and tissue remodeling. Cytokine signature profiles employed as diagnostic tools are discussed, together with the need for assay standardization. Advances in multiplex and omics technologies facilitating biomarker discovery are also reviewed. Finally, current and emerging therapeutic strategies targeting cytokines and growth factors, such as anti-TNF agents, IL inhibitors, anti-interferon therapies, and JAK/STAT pathway blockers, are explored.

## 1. Introduction

Cytokines are soluble proteins essential for coordinating and regulating the differentiation, maturation, and activation of immune cells through a network of interconnected signaling pathways. These proteins, secreted by different types of cells, bind to specific receptors on the surface of target cells to mediate diverse effects on cellular signaling and communication. An imbalance between pro-inflammatory and anti-inflammatory cytokines reflects immune dysregulation, which can trigger inflammatory responses and lead to tissue and organ damage [1]. In particular, autoimmune disorders occur when the immune system erroneously targets self-antigens, recognizing normal host tissues as foreign. This aberrant immune response results in chronic inflammation, tissue damage, and compromised organ function. Under normal conditions, the immune system discriminates effectively between self and non-self, facilitating the elimination of pathogenic microorganisms, including bacteria, viruses, parasites, and fungi, as well as malignant or aberrant cells, without initiating self-reactive responses. Immune system dysfunction can manifest in two principal forms: immunodeficiency, characterized by insufficient immune responses that fail to protect against infections, and autoimmunity, wherein immune tolerance is lost. The pathogenesis of autoimmune disorders fundamentally stems from a failure to maintain self-tolerance, leading to an inappropriate immune activation against endogenous tissues [2]. These conditions can be summarized under the name of autoimmune diseases (ADs). The etiology of ADs is multifactorial, involving a dynamic interplay of genetic predispositions, environmental exposures, epigenetic alterations, and hormonal influences. Genetic susceptibility, especially polymorphisms in human leukocyte antigen genes (HLA), is a predisposing factor for the development of autoimmune diseases, since these genes play an important role in the differentiation between self and non-self [3]. However, environmental factors such as infections, smoking, and long-term exposure to air pollution have also been shown to contribute significantly to disease development and exacerbation [4]. Rheumatoid arthritis (RA), systemic lupus erythematosus (SLE), psoriatic arthritis (PsA), Sjögren’s syndrome (SS), ankylosing spondylitis (AS), and systemic sclerosis (SSc) are only examples of autoimmune diseases, which are among the most common in the general population as well as the systemic autoimmune diseases for which there is more data available regarding the pathogenesis.

### 1.1. Rheumatoid Arthritis

RA is a chronic autoimmune condition that primarily targets the joints, leading to symptoms such as redness, swelling, and joint pain, and, in severe cases, to restricted mobility. Affecting about 1% of the population, RA is characterized by inflammation of the synovial membrane, which progressively leads to the degradation of cartilage and bone, ultimately resulting in joint deformity and chronic disability. Although the exact cause of RA is unknown, the immunological events occurring in the synovial tissue and synovial fluid have been well documented. Synovial macrophages are a key source of inflammatory cytokines such as TNF-α, IL-1, and IL-6, which drive inflammation, activate fibroblast-like synoviocytes (FLS), and enhance osteoclastogenesis, which contributes to bone erosion. Once activated, FLSs become aggressive cells capable of producing matrix metalloproteinases (MMPs), which degrade cartilage. Cartilage itself can release proteases in a feedback loop that further exacerbates damage [5,6].

### 1.2. Systemic Lupus Erythematosus

SLE is a multifaceted inflammatory autoimmune disorder marked by alternating phases of relapse and remission, during which multiple organs and systems can be affected. Key immunological features include the production of autoantibodies and activation of the complement system, resulting in the formation and deposition of immune complexes that contribute to tissue injury and multiorgan involvement. The differentiation and functional development of CD4^+^ T cell subsets, along with their cytokine secretion profiles, are aberrantly regulated, playing a pivotal role in the progression of the disease [7].

### 1.3. Psoriatic Arthritis

PsA is a heterogeneous, chronic, inflammatory immune-mediated disease characterized by musculoskeletal inflammation (arthritis, enthesitis, spondylitis, and dactylitis). It generally occurs in patients with psoriasis or a family history of psoriasis [8]. PsA has been demonstrated to be associated with uveitis and inflammatory bowel disease, including Crohn’s disease and ulcerative colitis. The name “psoriatic disease” was coined to reflect the pathogenesis of PsA, which is complex and multifaceted, involving an interplay of genetic predisposition, triggering environmental factors, and activation of the innate and adaptive immune system, although autoinflammation has also been implicated [9]. An additional systemic autoimmune disease is SS, primarily targeting the salivary and lacrimal glands. It manifests as dry eyes and dry mouth (sicca symptoms) and may be accompanied by systemic features such as arthritis, interstitial lung disease, and peripheral neuropathy. SS is characterized by the infiltration of glandular tissue by autoreactive lymphocytes, elevated type I interferon signatures, and B-cell hyperactivity, with a well-established association with anti-Ro/SSA and anti-La/SSB antibodies [10].

### 1.4. Ankylosing Spondylitis

AS is a prevalent form of chronic arthritis that primarily affects the axial skeleton (spine and sacroiliac joints) but can also involve peripheral joints like other spondyloarthritides, including psoriatic arthritis. AS is not a classic autoimmune disease: it does not have a female predominance and is not associated with MHC class II alleles, nor does it show significant autoantibodies or clinical response to drugs targeting T or B cells. It is therefore considered a hyperinflammatory disease driven by abnormal innate responses to tissue stress. Pathological features include chronic inflammation (synovitis, enthesitis, and osteitis), focal bone destruction, and pathological bone formation, which can lead to joint ankylosis. About a third of patients also have extra-articular manifestations, such as psoriasis, inflammatory bowel disease (Crohn’s, ulcerative colitis), and acute anterior uveitis [11].

### 1.5. Systemic Sclerosis

Finally, systemic sclerosis (SSc) is a rare and chronic autoimmune disease characterized by connective tissue involvement, a complex pathogenesis, and highly variable clinical manifestations. The disease arises from intricate interactions between genetic susceptibility and environmental triggers such as chemical exposures and infections. These factors cause endothelial injury, immune activation, and chronic inflammation, leading to the release of cytokines and growth factors like TGF-β that stimulate fibroblast activation and excessive collagen production, driving vascular damage and progressive cutaneous and internal organ fibrosis [12]. SSc is more common in females, typically appearing between ages 30 and 50. Genetic factors, including specific HLA and immune-related gene variants, heighten risk alongside environmental exposures. Mortality is relatively high, mainly due to lung complications. Early treatment targeting vascular, immune, and fibrotic pathways is essential to improve patient outcomes [13].

### 1.6. Cytokines and Growth Factors in Autoimmune Diseases

Despite their differences in clinical presentation, autoimmune and immunological-mediated disorders share a common underlying cause: immune system dysfunction. Chronic inflammation, abnormal immune cell activation, and overproduction of cytokines and growth factors all contribute to tissue damage and remodeling. Across these illnesses, cytokines and growth factors play critical roles in immunological dysfunction. Their dysregulation not only initiates and maintains inflammation, but it also causes structural alterations in tissues such as bone erosion, fibrosis, and glandular destruction. Understanding these molecular pathways is critical for designing targeted therapeutics capable of breaking the loop of immune-mediated harm and improving patient outcomes. Table 1 provides a detailed description of the main cytokines and growth factors involved in the pathogenesis of autoimmune diseases.

## 2. Pro-Inflammatory Cytokines in Autoimmune Diseases

### 2.1. Tumor Necrosis Factor Alpha (TNF-α)

#### 2.1.1. TNF-α Protein and Physiological Role

TNF-α is a multifunctional cytokine with a broad range of effects on different cell types. It plays a central role in regulating inflammatory responses and has been widely implicated in the development of various inflammatory and autoimmune diseases. Structurally, TNF-α is a homotrimeric protein composed of 157 amino acids and is primarily produced by activated macrophages, T lymphocytes, and natural killer (NK) cells. Functionally, TNF-α initiates the release of numerous inflammatory mediators, including other cytokines and chemokines. It exists in both a soluble form (sTNF-α) and a transmembrane form (tmTNF-α) [14]. To carry out its biological roles, TNF-α binds to two distinct membrane receptors: TNF receptor 1 (TNFR1) and TNF receptor 2 (TNFR2) [15]. sTNF-α primarily exerts its biological effects through interactions with TNFR1 and TNFR2. Many of TNF-α’s actions, such as cytotoxicity and regulation of cell proliferation, are mediated by TNFR1 activation. Depending on the cell type and surrounding environment, TNFR1 signaling can lead to a broad range of cellular outcomes, including cell proliferation, apoptosis, or necroptosis [16]. TmTNF-α also signals through both TNFR1 and TNFR2, although its effects are thought to be predominantly mediated via TNFR2. TNFR1 is ubiquitously expressed across nearly all human tissues and is considered the primary receptor responsible for the pro-inflammatory and cytotoxic effects of TNF-α. In contrast, TNFR2 is mainly found on immune cells, and an age-related imbalance between TNFR1 and TNFR2 activation may contribute to the age-related changes impacting both adaptive and innate immunity [17]. Binding of soluble TNF to TNFR1 initiates the formation of a receptor-ligand complex that recruits adaptor molecules such as TRADD, TRAF2, and RIPK1 to the receptor’s intracellular death domain. This leads to the formation of two signaling complexes: Complex I, which activates MAP kinase (MAPK), IκB kinase (IKK), and NF-κB pathways, driving cytokine and anti-apoptotic gene expression, and Complex II, which mediates apoptosis or necroptosis depending on caspase-8 activity. In contrast, TNFR2, activated primarily by membrane-bound TNF, lacks a death domain and directly interacts with TRAF2, TRAF1, and cellular inhibitors of apoptosis. TNFR2 signaling triggers MAPK, IKK/NF-κB, and PI3K/Akt pathways, promoting immune responses via activation of monocytes, macrophages, and T cells. The strength and nature of TNF responses are influenced by receptor expression levels, regulated by cytokines such as interferons, and the availability of intracellular adaptor proteins needed to assemble these signaling complexes [18]. During acute inflammation, TNF-α plays a major role in the acute phase response. It stimulates the production of C-reactive protein (CRP) and other acute-phase reactants. TNF-α also acts as an immunomodulator, enhancing the activity of innate immune cells. Furthermore, it can induce fever by acting on the hypothalamic–pituitary axis in coordination with other cytokines such as IL-1 and IL-6 [19]. However, while TNF-α is critical for normal immune function, its excessive or inappropriate production can lead to pathological consequences.

#### 2.1.2. TNF-α in Autoimmune Diseases

TNF-α plays a central role in the pathogenesis of RA. Its expression is significantly elevated in RA patients, and overexpression of TNF-α in transgenic animal models has been shown to induce autoimmune arthritis [20,21]. TNF-α signaling contributes to RA pathogenesis through multiple pathways. It activates endothelial cells and recruits various pro-inflammatory cells, including synovial fibroblasts and macrophages. These cells, in turn, release additional cytokines such as IL-6, IL-1β, and TNF itself, further amplifying the inflammatory response [22]. TNF-α also influences the differentiation and function of T helper 1 (Th1) and Th17 cells, promotes antibody production, and stimulates osteoclast differentiation, thereby contributing to bone erosion. Among the various cytokines involved, TNF-α is considered the primary inflammatory mediator in RA and is consistently found at elevated levels in affected individuals. The chronic inflammation in RA is marked by the accumulation of immune cells, mainly Th1 cells and macrophages, but also B cells, plasma cells, and dendritic cells (DCs) [23]. In RA, TNF-α secreted by Th1 cells and macrophages activates synovial fibroblasts, contributes to synovial hyperplasia, and facilitates the recruitment of additional inflammatory cells. Under the influence of cytokines such as IL-1, IL-6, and TNF-α, synovial fibroblasts upregulate cathepsins and MMPs, leading to the breakdown of collagen and proteoglycans. This cascade of events ultimately destroys cartilage and bone, culminating in joint erosion. Elevated TNF-α levels strongly correlate with disease activity and inflammation, reflecting the accumulation of Th1 cells, macrophages, B cells, plasma cells, and DCs in the affected tissues [24,25].

In systemic lupus erythematosus (SLE), dysregulation of TNF-α significantly contributes to the tissue damage that characterizes the systemic nature of the disease. TNF-α promotes lymphocyte apoptosis and impairs the clearance of apoptotic cells, leading to defective removal of cellular debris. This defective clearance results in the persistence of apoptotic remnants that expose modified or clustered self-antigens. Such exposure breaks immune tolerance by activating autoreactive lymphocytes, thereby promoting autoantibody production and amplifying autoimmune responses. Furthermore, the clustering of diverse intracellular antigens within apoptotic blebs facilitates a broad autoimmune response [26]. Indeed, in SLE patients, elevated TNF-α-induced apoptosis is associated with a rise in autoantibody levels, thereby enhancing disease activity [27]. However, the role of TNF-α in SLE is complex and somewhat controversial. While some studies suggest that TNF-α increases susceptibility to SLE [28], others have indicated that it may exert a protective effect in certain contexts. Elevated serum levels of TNF-α and its soluble receptors have been observed in patients with active SLE compared to those with inactive disease. Moreover, high TNF-α levels have been linked to increased disease severity [29,30]. Interestingly, one report indicated that TNF-α levels can be lower in patients with severe SLE, suggesting a possible protective role under certain conditions [31]. On the other end, higher TNF-α levels have been found in patients with acute disease compared to those without an acute cutaneous involvement, indicating that TNF-α may help modulate immune activity in specific disease stages [32].

TNF-α is also one of the abundant cytokines in inflammation related to SS; it is secreted by activated macrophages and T-cells and contributes to the expression of adhesion molecules and chemokines (e.g., IL-8), increasing the influx of other immune cells to the glandular tissue [33]. Indeed, in SS, TNF-α is highly expressed in damaged salivary glands, suggesting a direct role in glandular dysfunction and perpetuation of the autoimmune response [34]. TNF-α stimulates glandular epithelial cells to produce cytokines such as IL-6 and IL-8 and chemokines such as CXCL10, promoting the recruitment of T- and B-cells into the glandular tissue. In addition, it promotes the formation of ectopic lymphoid structures (TLS), which serve as local sites of immune activation [35]. TNF-α also contributes to epithelial dysfunction through activation of the NF-κB pathway, promoting oxidative stress and apoptosis of salivary epithelial cells [36].

TNF-α is also involved in the inflammatory process of AS. At the enthesis site (enthesis), it binds to TNFR1 and TNFR2 receptors on endothelial cells, osteoblasts, and osteoclasts, activating NF-κB and MAP kinase pathways. This results in a marked increase in the expression of cytokines (IL-1, IL-6), chemokines, and adhesion molecules (ICAM, VCAM), with a recall of neutrophils and other white blood cells into the joint [37]. TNF-α also promotes osteoclastic differentiation by increasing RANKL by osteoblasts, thereby promoting bone resorption [38]. In particular, TNF-α induces the expression of Dickkopf-1 (DKK-1), an antagonist of the Wnt pathway, altering the osteoblast/osteoclast balance and promoting the abnormal ossification typical of the disease [39]. In addition, TNF-α interacts with other mediators, in particular the IL-23/IL-17 axis: e.g., Th17 cells can amplify TNF-α production and vice versa, creating a self-feeding inflammatory circuit. TNF-α acts as a ‘switch’ in the immune-mediated inflammation of spondyloarthritis, making it a key therapeutic target [40].

Finally, in systemic sclerosis, TNF-α was found to be elevated in SSc patients, and its higher serum levels, influenced by specific TNFA gene promoter polymorphisms, correlate with an increased risk of cancer in these patients [41]. In addition, higher levels of TNF-α were observed in bronchoalveolar lavage (BAL) fluid from SSc patients with impaired lung function and greater pulmonary involvement [42].

### 2.2. Interleukin-1β (IL-1β)

#### 2.2.1. IL-1β Protein and Physiological Role

IL-1β is a pivotal pro-inflammatory cytokine and a key member of the IL-1 family, a group of 11 cytokines integral to orchestrating immune and inflammatory responses. Renowned for its potent inflammatory activity, IL-1β exerts profound effects on the pathogenesis of several chronic inflammatory systemic diseases such as Atherosclerosis, Metabolic Syndrome, Type 1 Diabetes and RA [43]. The synthesis and activation of IL-1β is tightly orchestrated by a multi-step process. This complex activation pathway is regulated at multiple levels to ensure precise modulation of its potent pro-inflammatory effects [44]. Its production is primarily triggered by the activation of inflammasomes, which are intracellular multiprotein complexes used as critical sensors of pathogenic and stress-related signals, initiating robust inflammatory cascades in response to extracellular threats. In particular, the NLRP3 inflammasome is activated by diverse cellular stress signals, including potassium efflux, mitochondrial dysfunction, and the generation of reactive oxygen species (ROS,) positioning it as a central player in inflammation-driven pathology [45]. This process causes the adaptor protein ASC to group together and attach, which then activates caspase-1 (also known as IL-1β-converting enzyme). Caspase-1 then cuts the inactive pro-IL-1β, turning it into its active, inflammatory form. Once released, IL-1β forms a signaling complex by binding to its receptor, IL-1 receptor 1 (IL-1R1), which triggers the activation of NF-κB and MAPK pathways, thereby amplifying the inflammatory response [46]. During inflammation, IL-1β plays a central regulatory role by orchestrating multiple key processes. It promotes the synthesis of collagenases and MMPs, drives lymphocyte activation and proliferation, and upregulates adhesion molecule expression, thereby facilitating endothelial cell activation. Furthermore, IL-1β elevates the levels of cyclooxygenase-2 (COX-2), C-reactive protein (CRP), and various protease inhibitors, collectively intensifying the inflammatory response and contributing to tissue remodeling and immune activation [47]. The amplification of inflammation following IL-1β activation is responsible for interactions with other pro-inflammatory cytokines, such as IL-8, which enhance Th1 and Th17 responses in autoimmune diseases and exacerbate tissue damage [48]. So, it is important to recognize that, although IL-1β is essential for host defense against infections, its production must be tightly regulated. Excessive or inappropriate activation can lead to chronic inflammation and tissue damage. To prevent this, negative feedback mechanisms are in place to limit IL-1β production, including the activation of anti-inflammatory pathways and the release of inflammasome inhibitors. However, when these regulatory mechanisms fail or are insufficient, dysregulated production or activity of IL-1β can contribute to the development of autoimmune or autoinflammatory conditions. Excessive IL-1β can promote a chronic inflammatory state that, over time, disrupts immune tolerance and leads to aberrant activation of the immune system against self-tissues. This dysregulation has been associated with various autoimmune diseases, in which IL-1β plays a key role in sustaining inflammation and driving tissue damage [49].

#### 2.2.2. IL-1β in Autoimmune Diseases

In RA, IL-1β is a pivotal mediator in both the initiation and perpetuation of joint inflammation and destruction. Its role extends beyond merely driving inflammation; IL-1β actively contributes to synovial hyperplasia by stimulating fibroblast-like synoviocytes (FLS) to proliferate and produce pro-inflammatory mediators [50]. These activated FLSs secrete MMPs, particularly MMP-1 and MMP-3, which degrade extracellular matrix components, including collagen, thus compromising cartilage integrity [51]. Additionally, IL-1β stimulates chondrocytes, contributing to cartilage degradation and promotes osteoclast activation, induced by the expression of receptor activator of nuclear factor kB ligand (RANKL), resulting in increased bone resorption [52].

Recent studies have demonstrated increased serum levels of IL-1β in SLE patients, correlating with disease activity and severity [53]. The production of IL-1β in SLE involves complex interactions between innate immune sensors and inflammatory pathways. It has been uncovered an unconventional mechanism where monocytes from SLE patients produce IL-1β through a pathway dependent on type I interferon (IFN) signaling. This process involves the internalization of mitochondria-containing red blood cells, leading to the activation of the NLRP3 inflammasome and subsequent IL-1β secretion without inducing cell death [54]. In addition, genetic studies have identified polymorphisms in the IL-1β gene associated with increased susceptibility to SLE. Specific IL-1β gene variants may influence disease risk and phenotype, highlighting the genetic contribution to IL-1β-mediated inflammation in SLE [55]. For instance, it has been found that the induction of LY6E, an IFN-stimulated gene, regulates IL-1β production, potentially contributing to the immunopathogenesis of SLE [56]. Additionally, IL-1β expression in cutaneous lupus erythematosus (CLE) skin biopsies has been associated with a tendency towards systemic involvement [57].

In recent years, IL-1β has received increasing attention for its key role in the pathogenesis of primary Sjögren’s syndrome [58,59]. Increased IL-1β production has been observed locally, in the minor salivary glands, as well as systemically, suggesting a multifactorial role in disease progression. IL-1β mRNA expression has been reported in the salivary glands of patients with SS [60]. Recent studies have reported that increased IL-1β in inflamed glands correlates with increased MMP-2 activity and decreased expression of its inhibitor TIMP-2, thus contributing to tissue damage and destruction of glandular structures [61]. Furthermore, an elevated presence of IL-1β has also been documented in peripheral blood and tears [62,63]. These findings suggest that inflammation in SS is not limited to the exocrine glands but also involves a systemic activation of innate immunity. The measurement of tear levels of IL-1β can contribute to the differential diagnosis between SS and other similar conditions, such as IgG4-related disease, with relevant diagnostic implications [64]. A further pathogenetic element involved in the regulation of IL-1β is related to the activation of the NLRP3 inflammasome [65]. Studies in mouse models and human glandular tissues have shown that activation of the P2 × 7 receptor stimulates the formation of the NLRP3 inflammasome, promoting the maturation and secretion of IL-1β by resident macrophages, thus contributing to the maintenance of chronic inflammation [66]. It has also been shown that IL-1β and IL-33 act synergistically in mast cell activation, amplifying the inflammatory response in salivary glands [67,68].

Recent studies have shown that IL-1β gene expression is significantly increased in patients with psoriatic arthritis compared to both healthy subjects and patients with cutaneous psoriasis alone. Notably, a 2022 study reported a more than 22-fold increase in IL-1β expression in PsA patients compared to controls, whereas the increase in patients with only cutaneous psoriasis was much more modest [69]. This suggests that IL-1β is probably involved in the transition from cutaneous psoriasis to psoriatic arthritis and also in the severity of joint inflammation. Indeed, the activation of the inflammasome, leading to IL-1β production, has been observed both in the peripheral blood and in the synovial fluid of PsA patients. The presence of elevated levels of active caspase-1 in synovial fluid further supports the central role of this cytokine in joint inflammation. Genetic polymorphisms in the IL-1β locus have been associated with increased susceptibility to the development of psoriatic arthritis, further supporting a direct pathogenetic role [70,71]. Although the IL-23/IL-17 axis is recognized as the dominant inflammatory pathway in PsA, IL-1β acts upstream and in synergy with other key cytokines such as TNF-α and IL-17, contributing to the activation of both innate and adaptive immune cells and to the perpetuation of chronic joint and skin inflammation [72].

IL-1β expression and activity are also elevated in SSc patients. Increased IL-1β levels have been observed in serum and bronchoalveolar lavage fluid of SSc patients [42,43,44,45,46,47,48,49,50,51,52,53,54,55,56,57,58,59,60,61,62,63,64,65,66,67,68,69,70,71,72,73]. Furthermore, IL-1β contributes to fibrosis by inducing activation and differentiation of fibroblasts into myofibroblasts, cells responsible for excessive extracellular matrix and collagen production, central to the fibrotic process in SSc. This occurs via pathways that include induction of IL-6, TGF-β1, and platelet-derived growth factor (PDGF), which are critical fibrosis mediators [74]. However, IL-1β also appears to exert an indirect activation effect on fibroblasts. Recent evidence demonstrates that IL-1β stimulates microvascular endothelial cells (MVECs) derived from SSc patients to promote the differentiation of monocytes into DCSIGN+CCL18^high^CCL2^high^CXCL8^high^IL-10^low^ alternatively activated macrophages. These macrophages, in turn, contribute to the activation of pro-inflammatory fibroblasts implicated in the fibrotic processes underlying scleroderma pathogenesis [75].

### 2.3. Interleukin-6 (IL-6)

#### 2.3.1. IL-6 Protein and Physiological Role

IL-6 is a small polypeptide that has a four-helix bundle structure and interacts with a membrane-anchored IL-6 receptor (IL-6R) present on target cells. It is produced by several cell types like B lymphocytes, T lymphocytes, macrophages, fibroblasts, keratinocytes, mesangial cells, vascular endothelial cells, mast cells, and dendritic cells, and it can be activated by IL-1β and TNF-α [76]. Upon binding, the IL-6/IL-6R complex recruits a second receptor subunit known as glycoprotein 130 (gp130). This receptor then forms a dimer, triggering downstream intracellular signaling cascades involving Janus kinases (JAKs), signal transducers and activators of transcription (STATs), the Ras/mitogen-activated protein kinase (MAPK) pathway, and the phosphoinositide 3-kinase (PI3K) signaling route [77]. Under physiological conditions, IL-6 contributes significantly to maintaining organ and cellular balance. Mice lacking the IL-6 gene tend to develop obesity later in life [78], are unable to regenerate liver tissue following partial hepatectomy [79], and do not exhibit osteoporosis after ovariectomy [80]. These observations suggest that IL-6 is involved in regulating body weight, supporting liver function, and maintaining bone health. In contrast, during disease states, notable distinctions emerge between IL-6 knockout and wild-type mice. IL-6-deficient mice show complete resistance in experimental models of rheumatoid arthritis and multiple sclerosis, underscoring the pivotal role of IL-6 in the development of these autoimmune diseases [81,82]. IL-6 is rapidly synthesized in response to pathogens or damage-associated molecular patterns (DAMPs) linked to inflammation. It plays a protective role by facilitating the elimination of infectious agents and promoting tissue repair through the activation of acute-phase responses and immune mechanisms. So, it is essential for the proper functioning of both innate and adaptive immune systems. Within the context of innate immunity, IL-6 facilitates the maturation of inflammatory infiltrates by promoting the recruitment of neutrophils and the infiltration of mononuclear cells. Additionally, it functions as a chemoattractant for monocytes at sites of inflammation. Regarding adaptive immunity, IL-6 exerts its effects on both T lymphocytes and B lymphocytes, influencing their activation and function [83].

This cytokine plays a regulatory role in the differentiation of monocytes into macrophages by modulating the production of macrophage colony-stimulating factor (M-CSF) [84]. Macrophages serve as key effector cells within the innate immune system and act as an early defense mechanism against invading pathogens. They engulf bacteria through phagocytosis and release antimicrobial molecules along with pro-inflammatory cytokines, amplifying the inflammatory response [85]. Additionally, macrophages can function as antigen-presenting cells for T lymphocytes and are involved in the removal of cellular debris from injured or dying cells via programmed cell death. IL-6 also influences the adaptive immune response by promoting Th2 cell differentiation and suppressing Th1 polarization. It stimulates CD4+ T cells to produce IL-4, which drives Th2 lineage commitment, while simultaneously reducing the production of interferon-gamma (IFN-γ), a cytokine essential for Th1 cell development [86].

#### 2.3.2. IL-6 in Autoimmune Diseases

In RA, IL-6 is highly expressed in synovial tissue and fluid, and its levels correlate with synovial inflammation and damage [87]. Synovial fibroblasts (FLS) and macrophages are the major sources of IL-6 in the joints of RA patients [88]. Activated FLSs secrete IL-6 (along with TNF-α, IL-1β, etc.) into the joint space, creating an inflammatory microenvironment. IL-6 from FLS stimulates STAT3 in infiltrating T cells and synovial cells, promoting an “aggressive” phenotype of FLS and osteoclast activation. Indeed, IL-6/sIL-6R trans-signaling enhances RANKL expression and osteoclastogenesis, driving bone erosion in arthritic models [89]. Furthermore, IL-6 induces vascular endothelial growth factor (VEGF) production by synoviocytes, promoting angiogenesis and pannus formation. RA-FLS secrete IL-6, fueling synovial hyperplasia and FLS invasiveness [90]. IL-6 (with TGF-β, IL-1β, IL-23) drives naïve CD4^+^ T cells into IL-17–producing Th17 cells [91]. In addition, this cytokine induces hepatic acute-phase reactants and VEGF, linking systemic manifestations (e.g., CRP elevation) with local joint inflammation [92]. IL-6 is elevated in many SLE patients, particularly those with active disease or lupus nephritis [93]. Monocytes/macrophages appear to be a principal IL-6 source in SLE [94]. At the molecular level, IL-6 promotes the hallmark B-cell hyperactivity of SLE: it drives B cells into plasma cells that secrete IgG autoantibodies as Ab anti-dsDNA [90]. IL-6 also supports T follicular helper (TFH) cells and skews the Th17/Treg balance toward pro-inflammatory Th17 in SLE, potentially amplifying tissue inflammation [95].

IL-6 is frequently elevated in serum, saliva, and tears of patients with SS [96]. Recent studies indicate that IL-6 is overexpressed in inflamed salivary glands, correlating with lymphocyte infiltrate and disease severity [97]. These studies also showed that IL-6, in combination with TGF-β1, induces epithelial–mesenchymal transition (EMT) in salivary epithelial cells, with loss of epithelial markers (E-cadherin) and acquisition of mesenchymal markers (vimentin), suggesting a direct role in fibrosis and tissue remodeling [98]. It promotes the differentiation of CD4+ T cells toward the Th17 phenotype, known for its role in chronic autoimmune inflammation. Th17 cells release IL-17, a cytokine that, together with IL-6, promotes epithelial damage and fibroblast activation. Moreover, IL-6 supports the maturation of B cells into plasma cells, promoting the production of autoantibodies such as anti-SSA/Ro and anti-SSB/La [99].

In AS, IL-6 is involved in the regulation of inflammation, T helper 17 (Th17) cell differentiation, and bone metabolism [100]. Recent studies have shown that serum levels of IL-6 are significantly elevated in patients with SA compared to healthy controls and correlate positively with disease severity [101]. In addition, IL-6 appears to contribute to osteoclast activation via RANKL induction and osteoblast inhibition, accentuating the imbalance in bone remodeling typical of AS [102].

In PsA, IL-6 contributes to the IL-17–driven inflammatory axis. Genetic and environmental triggers (dysbiosis, biomechanical stress) activate innate cells (dendritic cells, macrophages) to release cytokines (IL-12, IFN-α, IL-23, TGF-β, IL-6, IL-1β) that polarize T cells [103]. IL-6 (with TGF-β and IL-1β) promotes Th17 differentiation, leading to production of IL-17A/F, IL-22, and IL-26 [104]. Conversely, IL-6 blockade in PsA is generally ineffective, suggesting IL-6 is less dominant than TNF or IL-17 in PsA pathology. Nonetheless, IL-6 participates in key processes: it is produced by synovial fibroblasts, endothelial cells, and immune cells in psoriatic joints, and IL-6 levels rise in the synovium during inflammation. A hallmark of PsA is bone remodeling; here, IL-6 contributes to osteoclastogenesis. IL-17A (abundant in PsA) upregulates IL-6 in stromal cells and macrophages, which then drives osteoclast differentiation [105].

IL-6 serum levels correlate with disease severity features, including digital ulcers, calcinosis, anti-Scl70 antibodies (commonly associated with diffuse SSc), and impaired lung function. IL-6 is also linked with increased cardiovascular risk in SSc patients [106]. IL-6 plays a crucial role in systemic sclerosis (SS) by contributing to vascular damage and fibrosis development. It promotes collagen production through multiple pathways, including myofibroblast differentiation, inhibition of collagen-degrading matrix metalloproteinases, and activation of fibroblasts [107]. The IL-6 trans-signaling pathway, involving soluble IL-6 receptor (sIL-6R), is essential for driving collagen production and fibrotic changes, as demonstrated in murine models where IL-6 signaling caused SS-like symptoms such as pulmonary fibrosis and skin thickening [108]. Conversely, IL-6-deficient mice in bleomycin- induced lung fibrosis showed reduced pulmonary fibrosis and inflammation [109].

### 2.4. Interleukin-17 (IL-17)

#### 2.4.1. IL-17 Protein and Physiological Role

IL-17 is a pro-inflammatory cytokine that plays a central role in immune responses, especially at mucosal barriers. The IL-17 family comprises six structurally related cytokines (IL-17A-F), with IL-17A being the most studied and biologically significant member. While IL-17 is well known for its involvement in autoimmune and inflammatory diseases, its physiological functions are essential for host defense, tissue repair, and maintenance of barrier integrity [110]. IL-17 mediates its biological effects through the IL-17 receptor family, triggering signaling cascades such as NF-κB or MAPKs. The activation of these pathways leads to the production of pro-inflammatory cytokines, chemokines, and MMPs, which are essential for neutrophil recruitment and the amplification of inflammation in autoimmune diseases [111]. IL-23, a heterodimeric cytokine belonging to the IL-12 family, plays a pivotal role in Th17 cell-mediated immune responses by inducing the expression of IL-17A, IL-17F, IL-21, and IL-22. Furthermore, IL-23 promotes the polarization of activated T cells toward the Th17 lineage and stabilizes their phenotype, thereby enhancing their pro-inflammatory capacity. As previously noted, IL-23 functions as a critical mediator in the IL-17-driven crosstalk between innate and adaptive immunity. Inflammasomes regulate IL-17-associated responses by activating and releasing IL-1β, which is essential for Th17 cell differentiation, maintenance, and IL-17 production via IL-1/IL-1R signaling. The pro-inflammatory environment is intensified by the combined effects of IL-1β, IL-18, and IL-23, which enhance IL-17 expression in Th17 and γδ T cells. Additionally, IL-23 sustains IL-17 secretion and supports memory CD4^+^ T cell expansion, reinforcing the IL-23/IL-17 axis as a major driver of immune activation [112]. IL-17 is also crucial for defense against extracellular bacteria and fungi, especially at mucosal surfaces. Upon invasion by extracellular pathogens, IL-17s contribute to host defense in multiple ways. Firstly, they induce epithelial cells to produce antimicrobial peptides such as β-defensin and mucin, and they trigger the release of inflammatory mediators like prostaglandins and MMPs, which collectively form the body’s initial barrier against infection. Secondly, IL-17s strongly enhance the expression of pro-inflammatory cytokines like IL-6, IL-8, and TNF-α, as well as chemokines such as macrophage inflammatory protein-2, monocyte chemotactic protein-1, CXCL-8, CXCL-1, and CXCL10. These cytokines and chemokines orchestrate the recruitment of myeloid cells, especially neutrophils, to sites of infection or tissue injury, where they play a crucial role in pathogen recognition and clearance [113]. In addition, IL-17 contributes to tissue repair by modulating fibroblast activity and extracellular matrix production, inducing growth factors that facilitate wound healing, and regulating metabolic pathways in stromal cells (e.g., glucose uptake via CPT1A induction). These roles highlight IL-17’s importance in restoring tissue function following damage [114].

#### 2.4.2. IL-17 in Autoimmune Diseases

IL-17 is highly abundant in the synovial fluid and tissues of rheumatoid arthritis (RA) patients. It drives inflammation by stimulating the production of pro-inflammatory cytokines and matrix metalloproteinases (MMPs), which contribute to tissue degradation [115]. Additionally, IL-17 promotes osteoclastogenesis, leading to increased bone resorption and joint damage characteristic of RA [116]. Elevated levels of IL-17 have been observed in the peripheral blood and affected tissues of SLE patients [117]. IL-17 contributes to the pathogenesis of SLE by promoting the activation of dendritic cells, enhancing the survival of autoreactive B cells, and inducing the production of type I interferons [118]. Recent studies have indicated that IL-17 levels correlate with disease activity in SLE patients, suggesting its potential as a biomarker for disease monitoring [119].

IL-17 shows significantly increased expression in the salivary glands of SS patients, in association with the severity of local inflammation and systemic involvement [120]. Indeed, the IL-17/IL-23 axis appears to be particularly active in SS: lymphocytic infiltration in minor salivary glands is often characterized by an expansion of Th17 cells, accompanied by a local increase in IL-17 and IL-23 [41]. This axis promotes chronic inflammation and glandular destruction, exacerbating the exocrine dysfunction. One of the mechanisms through which IL-17 contributes to the progression of SS is the induction of epithelial–mesenchymal transition (EMT) in glandular epithelial cells, which leads to fibrosis and functional tissue loss [121]. This process is mediated by activation of the TGF-β1/Smad and TGF-β1/Erk1/2 signaling pathways. Recent studies have confirmed that IL-17 can directly activate these signaling pathways, promoting the expression of mesenchymal markers and reducing the integrity of glandular epithelium [122]. Experimental studies have shown that blocking IL-17 signaling (e.g., with an Adeno-IL17R:Fc vector) reduces lymphocyte infiltrate and enhances salivary secretion in mouse models of SS [123], confirming the pathogenetic importance of IL-17A. The interaction between IL-6 and TGF-β also promotes the differentiation of new Th17 lymphocytes, while the presence of IL-21 (produced by Tfh/Th17) stimulates B cells to mature into autoimmune antibody-producing plasma [124].

The onset of inflammation in AS results from a complex interaction of genetic, epigenetic, and environmental factors that disrupt immune regulation. The most significant genetic link is with the HLA-B27 allele of the MHC-I gene on chromosome 6. Specifically, in spondylitis, the abnormal expression of the HLA-B27 allele may contribute to the disease by binding to cells that express the natural killer receptor KIR3DL2, which recognizes the HLA-B27 homodimer. This interaction triggers the production of IL-17, a cytokine involved in driving inflammation in AS [125]. In this context, a strong connection between the gut and joints, known as the “gut-joints axis,” has emerged. Gut dysbiosis, increased IL-23 and IL-17 in the ileum, and impaired epithelial barrier suggest that the gut represents a central site in pathogenesis. Several mucosal immune cells, such as Th17, ILC3 (type 3 innate lymphoid cells), γδ T cells, and MAIT (Mucosal-Associated Invariant T), contribute to systemic and joint inflammation [126]. These cells, stimulated by IL-23, migrate to the inflamed sites by homing molecules such as integrin α4β7. Preclinical studies in HLA-B27 animal models have confirmed the central role of the microbiota, the IL-23/IL-17 axis, and Th17 cells in AS [127].

The IL-23/IL-17 axis plays a crucial role in the pathogenesis of psoriatic arthritis (PsA). Th17 cells produce the pro-inflammatory cytokine IL-17 and drive the expression of related molecules such as MMP3, CCL1, CCL20, and IL-6, all of which are significantly upregulated in the blood, synovium, and skin of PsA patients [128]. Additionally, CXCR6 has been identified as a marker for IL-17-producing CD8+ T cells in the synovial fluid of PsA patients, suggesting these specialized cells contribute to the inflammatory environment within the joints [129]. This highlights the central role of IL-17+ T cells in sustaining inflammation in PsA.

Finally, SSc patients with detectable IL-17A also showed increased levels of IL-1β, IL-6, and IL-22 and higher Th17 cell frequency. These patients tend to have reduced lung function and a significantly higher prevalence of pulmonary arterial hypertension (PAH) compared to patients without detectable IL-17A. Furthermore, SSc patients with both detectable IL-17A and high IL-6 levels exhibit more pronounced lung impairment and increased PAH prevalence than those with undetectable IL-17A and low IL-6 [130]. In early systemic sclerosis (SSc), inflammatory cells, including IL-17+ and Foxp3+ lymphocytes, infiltrate the skin. Patients with active SSc exhibit elevated percentages of circulating Th17 cells and increased IL-17 production, while Treg cell percentages remain unchanged. The number of Th17 cells strongly correlates with disease activity. IL-17 from SSc patients promotes fibroblast growth and collagen production, key drivers of fibrosis [131].

### 2.5. Interferon-Gamma (IFN-γ)

#### 2.5.1. IFN-γ Protein and Physiological Role

Interferons (IFNs) are a broadly expressed group of cytokines, classified into type I, II, and III based on the signaling receptors they engage [132]. The cytokine interferon-gamma (IFN-γ) is the sole member of type II interferons, and it plays an important role in the innate and adaptive immune responses [133]. IFN-γ initiates cellular responses by binding to its receptor, which leads to the activation of JAK1 and JAK2, two Janus kinases that associate with the receptor’s intracellular domain and become phosphorylated. This phosphorylation creates a docking site for signal transducer and activator of transcription 1 (STAT1), which is subsequently phosphorylated and forms homodimers [134]. Within normal immune function, IFN-γ contributes to the generation of reactive oxygen species, cytokine production, antigen presentation (including upregulation of MHC class II), regulation of metabolic processes, cellular differentiation, particularly macrophage polarization toward the pro-inflammatory M1 phenotype, and modulation of cell growth and survival [135]. Besides macrophages, IFNγ acts on various cell types, including leukocytes, vascular cells, adipose tissue cells, neurons, and tumor cells, influencing key processes related to autoimmunity [136].

#### 2.5.2. IFN-γ in Autoimmune Diseases

In the early stages of RA, IFN-γ contributes to the initiation and amplification of inflammatory responses [137]. It enhances antigen presentation by upregulating major histocompatibility complex (MHC) molecules and promotes the activation of macrophages and dendritic cells, leading to increased production of pro-inflammatory cytokines such as TNF-α and IL-1β. These actions facilitate the recruitment of immune cells to the synovial membrane, perpetuating inflammation and joint damage [138]. More recent studies demonstrated that plasma levels of IFN-γ were significantly elevated in patients with early RA compared to those with established RA and healthy controls, suggesting a role of this cytokine in the early stage of disease. Moreover, higher IFN-γ levels correlated with increased disease activity scores, suggesting its role in disease progression [139]. Contrary to its pro-inflammatory roles, IFN-γ also exhibits regulatory functions that can mitigate RA pathology. As an example, researchers highlighted that IFN-γ signaling could attenuate synovial inflammation by modulating the activity of FLSs and reducing the expression of MMPs, enzymes responsible for cartilage degradation [140].

In SLE, elevated levels of IFN-γ have been associated with disease activity and severity, contributing to the pathogenesis of SLE through the IFNGR1/2-pSTAT1-TBX21 signaling axis, promoting Th1 cell differentiation and activation, as well as B-cell complex formation, thereby exacerbating autoimmune responses [141]. Also, it has been evidenced that genetic variations in the IFN-γ gene have been implicated in SLE susceptibility. The +874 T/A polymorphism (rs2430561) has been associated with increased risk of SLE development, suggesting that individuals carrying the A allele may have a heightened risk of developing the disease [142].

IFN-γ represents, probably, the most highly expressed cytokine in patients with SS [143]. It activates macrophages and epithelial cells, increasing their expression of MHC molecules and promoting the inflammatory cascade [101]. SS patients have elevated levels of IFN-γ in tears and related tissues, in synergy with TNF-α, contributing to local inflammation [144]. Several studies have demonstrated the correlation between IFN-γ and salivary gland epithelial cell death (SGEC) via a mechanism of ferroptosis mediated by the JAK/STAT1 pathway. This process involves inhibition of the Xc-system and reduction in glutathione and GPX4, leading to oxidative damage and glandular dysfunction [145]. It has been identified that CD4^+^ T lymphocytes are a significant source of IFN-γ in the salivary glands of patients with SS, contributing to ferroptosis of SGECs and reduced expression of AQP5, a key protein for salivary secretion [146]. In patients with Sjögren’s syndrome (SS), overexpression of cell adhesion molecules (VACM-1, ICAM-1, PD-L1), regulated by IFNs via the JAK-STAT pathway, and influenced by oxidative stress, has been observed. In addition, recent studies suggest that type III IFNs produced by plasmacytoid dendritic cells (pDCs) also contribute to SS by promoting the maturation of pDCs and the pro-inflammatory immune response [147].

Several studies have documented a significant increase in IFN-γ levels in the peripheral blood of patients with AS compared with healthy controls. The finding suggests that IFN-γ may contribute to the maintenance of the chronic inflammatory state of the disease [148]. In some studies, it has been shown that certain polymorphisms in the IFN-γ gene, such as rs2430561, are associated with increased IFN-γ expression. Carriers of the T allele (TT genotype) show higher plasma levels of IFN-γ and thus an increased risk of contracting the disease [149].

IFN-γ plays a complex and dual role in the pathogenesis of PsA, with both therapeutic potential and pro-inflammatory effects observed across studies. IFN-γ induced localized psoriasis at injection sites and failed to normalize inflammatory biomarkers or improve cutaneous manifestations. Mechanistically, IFN-γ drives PsA progression through multiple pathways: elevated IFN-γ+ CD8+ T-cells correlate with disease activity in PsA scores and synovial inflammation. These cells synergize with IL-17A-producing Th17 cells to sustain joint damage; CXCL10 specifically associates with PsA development in psoriasis patients [150]. Contrasting with type I interferon (IFN-I) findings, PsA patients exhibit downregulated IFN-I signatures in peripheral blood mononuclear cells (PBMCs) despite elevated IL-6 and IL-1β expression. This dissociation suggests IFN-γ operates independently of IFN-I pathways in PsA pathogenesis. In addition, IFN-γ induces CXCL9-11 chemokines, recruiting monocytes, T-cells, and NK cells to the synovial fluid. Despite inhibiting RANKL-induced osteoclastogenesis via TRAF6 degradation, IFN-γ correlates with osteolytic lesions in PsA, suggesting indirect pro-resorptive effects through inflammatory mediators [151].

A large transethnic meta-analysis of GWAS, including 4436 SSc cases and 14, 751 controls, identified a significant association with the SNP rs4134466 in the Blimp-1 gene, which regulates IFN-γ gene expression [152]. Additionally, several polymorphisms in interferon regulatory factor (IRF) family genes—IRF5, IRF7, and IRF8—have been linked to SSc susceptibility and clinical features. IRF5 shows one of the strongest associations with SSc; certain IRF5 SNPs correlate with disease severity and survival, with higher IRF5 expression linked to worse lung disease. IRF8 variants are associated with increased IFN-γ expression and modulation of immune signaling pathways that bridge innate and adaptive immunity, including toll-like receptor (TLR) signaling [153].

## 3. Anti-Inflammatory Cytokines as Biomarkers in ADs

### 3.1. Interleukin 10 (IL-10)

#### 3.1.1. IL-10 Protein and Physiological Role

IL-10 is a cytokine that plays a key role in regulating immune responses, and it is produced by activated immune cells [154]. It is a major stimulant of B cells and has recently been identified as a critical driver of the extrafollicular B cell response [155]. Its receptor is a transmembrane protein composed of two subunits: IL-10R1 and IL-10R2 [156]. Initially discovered, IL-10 was named the Cytokine Synthesis Inhibitory Factor (CSIF) thanks to its ability to inhibit the activation and cytokine production of T helper (Th)1 cells. Although originally associated with the Th2 cytokine profile, it was later found that IL-10 is also produced by late-stage Th1 cells [157]. One of the most important functions of IL-10 is to suppress excessive pro-inflammatory responses that could lead to tissue damage. Consistent with this role, IL-10 inhibits the upregulation of MHC class II molecules and costimulatory markers on antigen-presenting cells (APCs), thereby reducing the production of inflammatory cytokines [158]. Furthermore, IL-10 helps prevent inappropriate T cell activation and proliferation, which could otherwise result in inflammatory effector responses. IL-10 secreted by plasma cells has localized effects on nearby neutrophils and myeloid cells. Overall, IL-10 production by both B and T cells plays a significant role in maintaining immune suppression [159]. Although most hematopoietic cells are capable of sensing IL-10 through the expression of the IL-10 receptor (IL-10R), recent studies have identified macrophages as the primary target cells of IL-10’s inhibitory effects [154]. Macrophages are key components of the innate immune system, playing a central role in orchestrating both innate and adaptive immune responses to various pathogens. They detect pathogens and tissue damage, act as antigen-presenting cells to initiate adaptive immunity, drive inflammation and host defense mechanisms, and contribute to tissue repair [160]. When exposed to classical inflammatory stimuli such as lipopolysaccharide (LPS) and interferon-γ, macrophages polarize toward the M1 phenotype. M1 macrophages are involved in host defense against bacterial, viral, and protozoal infections and also contribute to antitumor immunity. In contrast, exposure to alternative stimuli such as IL-10 and transforming growth factor-beta (TGF-β) promotes polarization toward the M2 phenotype [161].

#### 3.1.2. IL-10 in Autoimmune Diseases

In RA, this persistent activation of PRRs leads to continuous release of pro-inflammatory cytokines, contributing to the autoimmune pathology and joint damage. Regulatory mechanisms, especially involving the anti-inflammatory cytokine IL-10, act to limit this process by suppressing key adaptor molecules like MyD88 and promoting degradation of signaling proteins such as TRAF6 [162]. In experimental models of antigen- induced arthritis, IL-10 has shown protective effects, and mice lacking IL-10 exhibit exacerbated joint inflammation [163]. In RA patients, IL-10 is abundantly present in the synovial fluid [164] and has also been implicated in the regulation of bone resorption, primarily through its inhibitory effects on osteoclastogenesis in vitro [165].

Despite its well-established anti-inflammatory properties, the role of IL-10 in SLE remains controversial. While some studies report a positive correlation between IL-10 levels and disease activity or damage indices, others do not support this association [166]. IL-10 is generally considered protective in autoimmune diseases due to its ability to suppress pathogenic inflammation and promote immune tolerance. It mitigates inflammation by reducing the production of pro-inflammatory cytokines, diminishing the antigen-presenting capacity of immune cells, and inhibiting T helper cell functions [167].

In SS, IL-10 production appears to be impaired: studies in patients and mouse models have found reduced levels of IL-10 and CD4+IL-10+ T cells [168]. On the other hand, healthy Treg lymphocytes release IL-10 (in addition to IL-35) to inhibit Th17pmc cell differentiation [169]. Similarly, B Regulatory (Breg) cells secreting IL-10 can repress follicular helper T (Tfh) cell responses, reducing the generation of autoantibodies [170]. However, it appears that as SS disease progresses, the frequency of Breg-IL-10 decreases, while that of Tfh increases, translating into less regulatory control. In summary, IL-10 deficiency promotes an uncontrolled inflammatory framework in SS.

IL-10 gene promoter polymorphism-1082 A>G (rs1800896) was found to occur more frequently in AS patients, with individuals carrying the A/G or G/G genotypes having about three times higher risk of developing the disease compared to healthy controls. Furthermore, AS patients exhibit elevated serum levels of IL-10, indicating dysregulation of IL-10 production in the disease. These findings suggest that genetic variations in the IL10 gene influence both susceptibilities to AS and the systemic expression of IL-10, which likely affects the modulation of inflammatory and immune responses in AS [171].

### 3.2. Interleukin-4 (IL-4)

#### 3.2.1. IL-4 Protein and Its Physiological Role

IL-4 is a critical cytokine produced mainly by Th2 cells that regulates B cell differentiation, survival, and memory formation, particularly by modulating germinal center B cells through downregulation of the transcription factor BCL6. This regulation must be precisely balanced, as both excessive and impaired IL-4 signaling can disrupt memory B cell development, leading either to failed differentiation or apoptosis without proper survival signals [172]. Transcriptomic analyses have revealed that IL-4 influences the expression of genes associated with memory B cell differentiation. This suggests that IL-4 may suppress MBC differentiation under certain conditions, potentially modulating the balance between plasma cell and memory B cell fates [173]. In addition, IL-4 plays a pivotal role in tissue repair and the resolution of inflammation [174]. Beyond its classic role in promoting Th2 differentiation and immunoglobulin class switching, IL-4 is now recognized as a fundamental cytokine in orchestrating the healing process following tissue injury, both in acute and chronic settings. A central mechanism by which IL-4 contributes to tissue repair is through the polarization of macrophages toward the M2 (alternatively activated) phenotype. IL-4 acts directly on macrophages to upregulate markers like arginase-1, YM1, Fizz1, and mannose receptor (CD206), facilitating a wound-healing response [175]. This regulatory function of IL-4 is especially important in epithelial-rich tissues such as skin, lung, and gut, where rapid resolution of inflammation is essential to restore barrier function. Chronic IL-4 signaling leads to sustained M2 macrophage activation, which in turn stimulates excessive ECM deposition and fibroblast activation, potentially leading to scarring and organ dysfunction [176]. The role of IL-4 in autoimmune diseases is complex and context-dependent. While it supports Treg function, IL-4 can also influence the differentiation of other T cell subsets. For instance, IL-4 has been shown to inhibit the differentiation of Tregs through the regulation of CD103+ dendritic cells, which are involved in the induction of Treg differentiation. This suggests that IL-4’s effects on Tregs may vary depending on the local immune environment and the presence of other cytokines [177].

#### 3.2.2. IL-4 in Autoimmune Diseases

Recent research underscores IL-4’s dual role in rheumatoid arthritis (RA) pathogenesis and therapeutic potential. A critical function of IL-4 is its suppression of pro-inflammatory cytokines and inhibition of Th1 and Th17 cell differentiation, key drivers of RA pathology. For example, IL-4 reduces inflammatory activity by lowering IL-1β, TNF-α, and IL-6 levels in synovial macrophages and fibroblasts [178]. IL-4 also modulates neutrophil behavior. It has been demonstrated that IL-4 enhances the inhibitory FcγR2b receptor on neutrophils and curtails their migration into inflamed joints, mitigating tissue damage. This highlights IL-4’s role in regulating innate immune responses in RA [179].

In the context of SLE, IL-4 plays a critical and supportive role in sustaining the immunosuppressive functions of Treg cells. IL-4 significantly enhances the suppressive activity of Tregs. This is particularly important in SLE, where immune dysregulation is prominent, and Treg dysfunction contributes to the loss of peripheral tolerance and the expansion of autoreactive lymphocytes [180]. In IL-4-deficient mice, regulatory T cells (Tregs) show severely reduced suppressive capacity, resulting in increased activation of conventional T cells in OVA- sensitized animals. Supplementation with IL-4 restores Treg function by enhancing their survival in inflammatory environments, increasing granzyme expression for cytotoxic suppression, and improving their ability to inhibit responder T cell proliferation. These findings suggest that IL-4 is essential for the homeostatic maintenance of Treg-mediated immune tolerance, which is severely compromised in autoimmune conditions like SLE.

An analysis of cytokine levels in tears from patients with SS showed a significant increase in IL-4 compared with healthy controls and patients with dry eye not associated with SS. These data suggest that IL-4 might contribute to local inflammation and glandular dysfunction in SS [181].

More insights come from studies in PsA, where emerging evidence positions IL-4 as a disease-modulating cytokine influencing both inflammatory pathways and bone-related immune processes. IL-4 has been identified as a potential PsA-specific biomarker, with strong correlations to genes governing immune regulation and osteoclast differentiation [182]. Mechanistic studies reveal IL-4 reduces pro-inflammatory cytokines like IL-1β and IL-6 in psoriatic skin cells while boosting GATA3, a transcription factor driving Th2 cell development and inflammation resolution. This dual action implies IL-4 helps restore immune balance and prevent tissue destruction in psoriatic conditions [183]. IL-4 also suppresses Th17 cell activity, a major contributor to PsA. Researchers indicate IL-4 blocks IL-23 production in antigen-presenting cells, disrupting the survival and differentiation of pathogenic Th17 cells. This interaction is particularly significant in PsA, where the IL-23/IL-17 pathway fuels both cutaneous and articular damage [184].

## 4. Growth Factors as Biomarkers in ADs

### 4.1. Transforming Growth Factor Beta (TGF-β)

#### 4.1.1. TGF-β Protein and Physiological Role

TGF-β is a key and multifaceted regulator of immune responses, first identified over thirty years ago as a modulator of immune cell function. TGF-β influences both the intensity and nature of immune reactions to microbial threats and plays a crucial role in preserving immune tolerance and maintaining homeostasis in response to self-antigens and harmless environmental antigens under steady-state conditions [185]. While TGF-β is widely recognized for its role in promoting immune tolerance in peripheral tissues, its influence on T-cell biology extends far beyond its immunosuppressive effects. In fact, TGF-β is also essential for the development and differentiation of various T-cell subsets [186]. TGF-β exists in three different forms—TGF-β1, TGF-β2, and TGF-β3—and almost all cells in the body have receptors that can respond to these molecules. Among them, TGF-β1 is the most produced by cells of the immune system [187].

#### 4.1.2. TGF-β in Autoimmune Diseases

In a recent systematic review and meta-analysis [188], it was shown that certain polymorphisms in the TGF-β1 gene, such as the T869C variant, which in turn changes its signal peptide and secretion level [189], have been associated with increased RA susceptibility. Indeed, TGF-β can contribute to the pathological process by promoting the migration and invasion of fibroblast-like synoviocytes via the TGF-β1/Smad signaling pathway, leading to joint destruction [190].

In SLE, TGF-β signaling is often impaired. Studies have shown that the expression of TGF-β type I receptor (TGFβRI) is decreased in naïve CD4+ T cells of SLE patients, correlating with disease activity. Elevated levels of IL-6 in SLE patients can downregulate TGFβRI expression through the JAK/STAT3 pathway, further disrupting TGF-β signaling. This impairment may contribute to the loss of immune tolerance and the development of the autoimmunity characteristic of SLE [191].

A particular relevance to the pathogenesis of AS concerns the interaction between TGF-β and HLA-B27. Studies have shown that HLA-B27 expression alters the TGF-β signaling pathway, involving a physical interaction between HLA-B27 and the TGF-β type I receptor (ALK5). This interaction leads to increased phosphorylation of SMAD2/3, a key molecule in the downstream signal transduction of TGF-β, in B27 rat T cells. This alteration could contribute to the abnormal expansion of pro-inflammatory T helper 17 (Th17) cells and an altered phenotype of T-regs [192].

In SSc, abnormal activation of TGF-β is a signature of the disease, leading to excessive stimulation of fibroblasts and increased production of extracellular matrix components, causing the characteristic tissue fibrosis in the skin and internal organs [193]. Additionally, TGF-β influences the vascular system by affecting endothelial and smooth muscle cells, linking it to the vascular complications such as pulmonary arterial hypertension often seen in SSc [194]. The cytokine also modulates immune responses by regulating T cell activity and bridging inflammation with fibrotic and vascular changes [195]. Elevated levels of TGF-β have been found in the blood and tissues of patients with systemic sclerosis, underscoring its important role in the disease’s pathogenesis [196].

### 4.2. Other Growth Factors Involved in Autoimmune Diseases

Granulocyte-macrophage colony-stimulating factor (GM-CSF) drives macrophage polarization in rheumatoid arthritis (RA), contributing significantly to disease pathology [197]. Elevated baseline GM-CSF levels in RA patients have been identified as independent predictors of suboptimal or poor response to anti–IL-6 receptor (IL-6R) therapy [198]. GM-CSF is widely recognized as a key inflammatory mediator that promotes immune cell activation and sustains chronic inflammation in RA [199]. Another growth factor relevant in this context is the vascular endothelial growth factor (VEGF), which is a key driver of synovial angiogenesis, promoting endothelial cell proliferation under hypoxic and inflammatory conditions [200]. It induces RANKL expression in synovial fibroblasts, enhancing osteoclast differentiation and subsequent bone erosion [201]. Furthermore, elevated VEGF levels in serum and synovial fluid correlate with RA disease activity and joint damage [202]. VEGF is also a molecular driver in PsA disease, contributing to the inflammation and joint symptoms seen in PsA, such as pain and stiffness. Circulating VEGF also correlates positively with disease activity indicators, while treatments that control disease activity tend to reduce VEGF levels, highlighting VEGF’s potential as a biomarker for monitoring disease progression and response to therapy [203].

**Figure 1:**
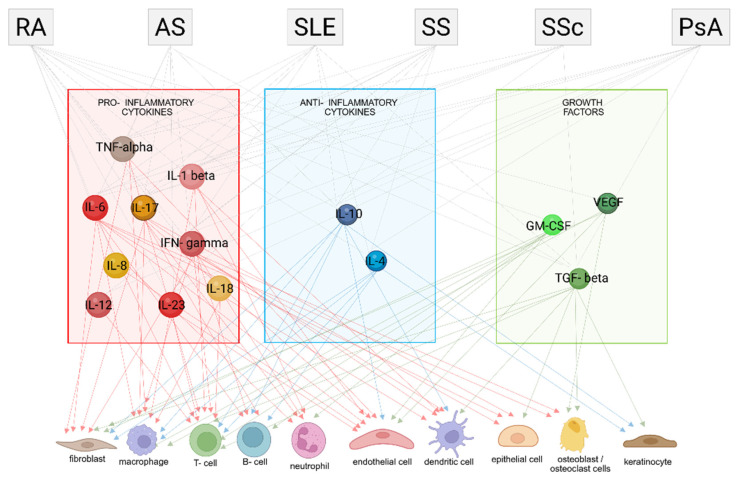
Cytokine networks and cell targets in autoimmune diseases: pro-inflammatory, anti-inflammatory cytokines and growth factors. Rheumatoid arthritis (RA), Ankylosing Spondylitis (AS), Systemic Lupus Erythematosus (SLE), Sjögren’s Syndrome (SS), Systemic Sclerosis (SSc) and Psoriatic Arthritis (PsA). Figure partly created in BioRender. Rosati, A. (2025) https://BioRender.com/lrjlo8t (accessed on 7 July 2025).

Figure 1 illustrates the intricate networks of cytokines and growth factors, along with their respective cellular targets, involved in the pathogenesis of autoimmune diseases. These molecular interactions, which have been thoroughly examined in the preceding three paragraphs, highlight the dynamic crosstalk between immune signaling pathways and specific cell populations.

## 5. Challenges in Biomarker Development

Cytokines and growth factors are central to immune regulation and tissue repair, making them attractive biomarkers for disease diagnosis, prognosis, and therapeutic monitoring. However, their inherent biological variability, such as short serum half-lives, low baseline concentrations, and context-dependent expression, complicates reliable measurement. For instance, cytokines involved in inflammation and immune response often fluctuate rapidly and may lack tissue or toxicity specificity, limiting their predictive power for certain conditions [204]. Analytical challenges include assay variability, cross-reactivity in multiplex immunoassays, and sensitivity differences that affect reproducibility and comparability across studies [205]. One of the primary challenges is the inherent variability in cytokine and growth factor levels across different individuals. This variability can arise from a multitude of factors, including genetic differences, environmental exposures, and disease-specific characteristics. In particular, environmental factors such as diet, the composition of the gut microbiota, and infections can all influence immune responses and consequently alter cytokine production [206,207].

The absence of standardized protocols for biomarker discovery and validation remains another significant barrier. Variability in sample handling, assay platforms, and data interpretation undermines the reliability of cytokine and growth factor measurements, hindering regulatory approval and clinical adoption [208]. Also, cytokines often lack tissue-specific or toxicity-specific expression, which contributes to this overlap and can reduce the specificity of individual cytokines as diagnostic biomarkers [209]. This necessitates the development of multi-cytokine panels or the identification of more disease-specific markers to improve diagnostic accuracy and differentiation between various disease conditions [210]. In addition, variability in sample processing techniques, storage conditions, and the specific methodologies employed in different assays can significantly affect the measured levels of cytokines and growth factors [211]. Innovative approaches are addressing these challenges by integrating multiplex and multi-omic technologies, such as genomics, proteomics, and spatial proteomics, to enhance biomarker specificity and sensitivity [212].

Multiplex technologies could greatly enhance the clinical management of autoimmune diseases by enabling simultaneous measurement of multiple immune markers, capturing disease complexity more effectively than single biomarkers [213]. This allows for improved diagnosis, prognosis, and treatment monitoring. However, their clinical adoption depends on strict standardization of sample handling and advanced data analysis tools to ensure reliable, interpretable results. When integrated properly, these assays could significantly improve personalized care in autoimmune conditions.

## 6. Therapeutic Implications

**Figure 2:**
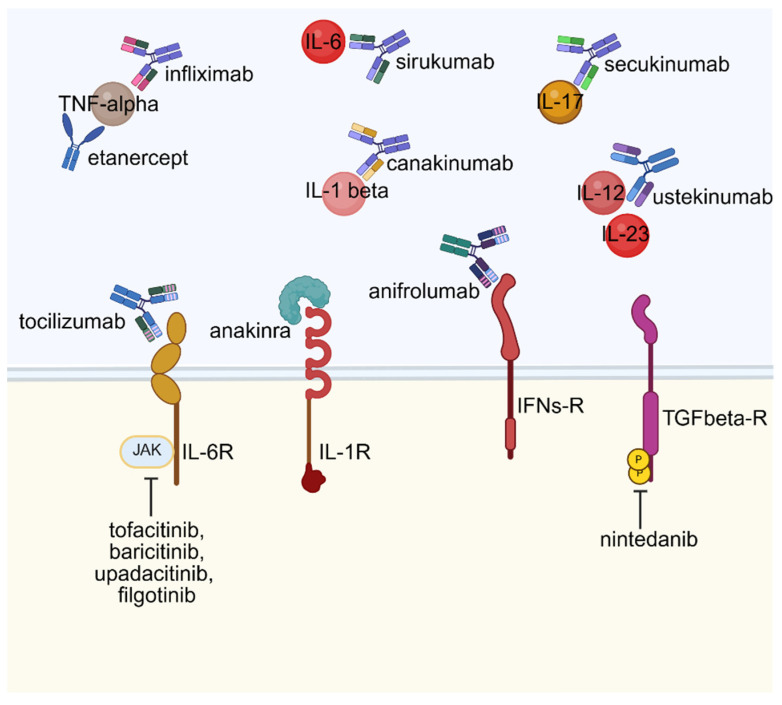
Targeted biologic and small molecule therapies in cytokine signaling pathways. Created in BioRender. Rosati, A. (2025) https://BioRender.com/kq4e7gh (accessed on 22 July 2025).

Therapeutic strategies targeting cytokines and growth factors have become essential in the management of autoimmune and inflammatory diseases due to the critical roles these molecules play in regulating immune responses and maintaining tissue homeostasis. Advances in our understanding of their complex biology, alongside the development of precise targeted therapies, have significantly improved clinical outcomes for patients with various autoimmune conditions. These therapeutic agents can be broadly classified into three categories based on their mechanisms of action: those that act on soluble signaling molecules by preventing or potentiating their activity, those that modulate cytokine or growth factor receptor activation, and those that interfere with intracellular signaling pathways—such as kinase inhibitors—that disrupt downstream signal transduction (Figure 2). This clear mechanistic framework guides the development of targeted treatments that achieve precise modulation of immune activity, offering more effective and tailored approaches in autoimmune disease therapy.

### 6.1. Targeting Secreted Immune Mediators in Autoimmune Therapy

Pro-inflammatory cytokines play pivotal roles in driving autoimmune inflammation and consequent tissue damage. Therapeutic strategies targeting these cytokines have revolutionized treatment paradigms for autoimmune diseases, particularly rheumatoid arthritis (RA). TNF inhibitors, exemplified by infliximab, disrupt cytokine-mediated signaling cascades, effectively reducing inflammation and improving clinical symptoms. Their efficacy is well-established through numerous clinical trials, leading to their widespread recommendation as first-line therapies in rheumatoid arthritis [214], psoriatic arthritis (PsA) [215], and ankylosing spondylitis (AS) [216] according to clinical guidelines. Nonetheless, despite TNF-α’s implicated role in Sjögren’s syndrome (SS) pathogenesis, anti-TNF therapy has yielded controversial and inconsistent results. Infliximab demonstrated initial promising functional outcomes in pilot studies; however, larger trials failed to confirm a significant clinical benefit [217]. Similarly, etanercept, another TNF-α blocker, showed no improvement in sicca symptoms or histopathology in SS patients [218]. Despite the absence of randomized controlled studies examining the effects of anti-TNF drugs on patients with SSc or SSc-ILD, observational studies conducted in the early 2000s indicated that anti-TNF drugs, especially infliximab and etanercept, may improve inflammatory arthritis and disability in patients with SSc and SSc-associated interstitial lung disease (SSc-ILD) [219].

Regarding systemic lupus erythematosus (SLE), anti-TNF therapy is rarely used due to the potential induction of lupus-like syndromes, although etanercept has occasionally been employed with some success for refractory arthritis in clinical observational studies [220]. In this context, the efficacy of anti-IL-6 treatments has also been assessed in SLE patients. In particular, sirukumab, an anti-IL6 monoclonal antibody, has been used in active lupus nephritis; however, without good results in terms of reduction in proteinuria [221].

Another target is IL-17, produced mainly by Th17 cells, which is foundational in the pathogenesis of several autoimmune diseases, including psoriasis, PsA, and AS.

IL-17 blockade, via agents like secukinumab and bimekizumab [222], has demonstrated significant efficacy in improving joint symptoms, both peripheral and axial, and skin lesions in PsA; they are considered effective treatment for PsA and AS [223].

In SS, IL-17 seems to have a role in the first phases of the disease, since IL-17 was found highly expressed in minor salivary glands of patients with disease duration less than 10 years, but not in their serum [224].

Growing evidence supports their potential use in SS, with clinical studies investigating effects on sicca symptoms and dry eye manifestations (CTI: NCT01250171).

A potential role of direct inhibitors of IL-17 in lupus nephritis has been suggested, since Th17 cells are involved in SLE pathogenesis as combination therapy, and after some successful case reports described in the literature, a phase III RCT to evaluate secukinumab in patients with active lupus nephritis has been conducted (CTI: NCT04181762). The study now is terminated, but no positive results have been published.

Although strong preclinical rationale supports IL-17 as a therapeutic target in RA, clinical trials provide mixed data; some demonstrate efficacy in biologic-naïve patients or those unresponsive to TNF inhibitors, whereas others indicate limited therapeutic impact, suggesting that IL-17 is not the sole pathological mediator in RA inflammation [225].

Also, IL-12/IL-23 are involved in the IL-17 activation pathway. Ustekinumab, a human monoclonal antibody targeting IL-12/IL-23, was approved for the treatment of psoriatic arthritis in 2013, but it has shown favorable outcomes in a phase II randomized controlled trial for patients with active lupus [226], and it has been reported to be effective for joint inflammation in patients with SS and RA, although there is still a lack of larger clinical trials [227].

Tildrakizumab, risankizumab, and guselkumab are IL-23p19 inhibitors, effective for the treatment of psoriasis and psoriatic arthritis [228]. The efficacy of this class of drugs in treating axial inflammation is unclear, and they usually are not considered as a therapeutic option in case of axial involvement.

Novel monoclonal antibodies against IL-1β, such as canakinumab, are under clinical evaluation, showing promising modulation of disease activity and favorable safety profiles [229]. Like anakinra, discussed later, canakinumab has also been shown to be effective for the treatment of rheumatoid arthritis; however, given the numerous therapeutic alternatives already existing, it has not been approved for this indication [230].

Immunotherapeutic strategies are increasingly focusing on enhancing regulatory cytokines such as IL-10. Studies conducted on recombinant human IL-10 (rhIL-10) for inflammatory bowel disease (IBD) have shown weak and inconsistent efficacy, partly attributable to its short half-life and the presence of pro-inflammatory properties that may counterbalance its beneficial effects [231]. New approaches, such as antibody grafts of IL-10, are being explored to improve cellular selectivity and prolong the half-life of the cytokine, seeking to overcome the limitations of previous formulations [232]. To date, no IL-10–centric treatment has yet been approved for AS or other autoimmune diseases. In this trajectory, regulatory B cells (Bregs) secreting immunosuppressive cytokines and exosome-based approaches exhibit promising results in preclinical models of central nervous system autoimmunity, aiming to restore immune homeostasis without broad immunosuppression [233].

### 6.2. Targeting Cell Surface Receptors in Autoimmune Therapy

Tocilizumab is an anti-IL-6 receptor antagonist and is approved in several countries for the treatment of rheumatoid arthritis, thanks to clinical trials and real-world data that confirm its efficacy from both a clinical and radiological point of view [234]. The inhibition of the same pathway has a positive effect also in other inflammatory rheumatic diseases like large vessel vasculitis and Still’s disease, while it has no therapeutic effect for the treatment of the other chronic inflammatory arthritides like psoriatic arthritis and ankylosing spondylitis [235].

Among connective tissue diseases, the most consistent data regarding the efficacy of IL-6 pathway inhibition came from the FocuSSced trial, in which tocilizumab or placebo was administered to patients with SSc [235].

Although the primary endpoint of the study, which was the improvement of skin fibrosis, was not met, it was noted that patients treated with tocilizumab had an improvement and stabilization of respiratory outcomes in the context of systemic sclerosis interstitial lung disease [236]. Based on these results and several case reports and case series described in the literature, tocilizumab has been included in the most recent guidelines among the therapeutic options for patients with systemic sclerosis and pulmonary or skin involvement, although with different degrees of evidence [237].

Considering the integral role of this cytokine in SS, the drug has also been tested in a randomized controlled trial in patients with SS. Nevertheless, tocilizumab did not improve systemic involvement and symptoms during treatment [238]. Conversely, tocilizumab was evaluated in a phase I open-label study in patients with moderately active SLE with significant improvement of disease activity scores SLEDAI and SLAM [239]. Despite these positive results, no randomized studies of more advanced phases have been conducted with this drug, and only case reports are available in the literature, with successful results especially in patients with refractory serositis and arthritis flares [240].

Clinical and preclinical studies have also underscored the therapeutic potential of IL-1β blockade in rheumatic disease. Anakinra, an IL-1 receptor antagonist, has demonstrated clinical efficacy in reducing joint inflammation and slowing structural damage in RA patients, while showing no significant benefit in psoriatic arthritis [241]. In patients with SS, IL-1 resulted in augmentation in salivary fluid, tears, and cerebrospinal fluid. At the glandular level, IL-1 acts through both direct damage on the glands and through inhibition of the neurotransmitter release in the sympathetic nervous system. Despite the role of IL-1 in SS, there is only one double-blind randomized clinical trial that has investigated the use of anakinra in the treatment of SS. The primary endpoint has not been reached, but in a post hoc analysis a reduction of 50% in fatigue was experienced in the group of patients treated with anakinra, suggesting a possible role of this drug in treating this kind of manifestation [242].

Anti-interferon α receptor monoclonal antibody anifrolumab targets the IFN-α receptor and has shown promise in SLE following TULIP I and II trials, impacting multiple type I and II IFN-induced gene modules/pathways and type III IFN-λ protein levels [243]. The results of these trials were so encouraging that anifrolumab was included in the latest treatment guidelines for SLE as add-on therapy in moderate to severe disease, especially in patients with severe skin disease where the interferon pathway seems to be most involved [244].

The presence of an IFN signature has been observed also in other connective tissue diseases like Sjogren’s syndrome, systemic sclerosis, and a subset of patients with rheumatoid arthritis, so multiple trials targeting this pathway are starting to recruit or have already recently started.

A pivotal Phase III study, referred to as the DAISY trial (CTI: NCT05925803), is now underway to evaluate the efficacy and safety of anifrolumab in adults with systemic sclerosis. An estimated 306 participants with either limited or diffuse cutaneous SSc—diagnosed according to the 2013 ACR/EULAR classification criteria—will be enrolled from multiple international centers.

Moreover, a phase 2 multicenter study, TarIFNiRA (CTI:NCT03435601), to evaluate the efficacy and safety of anifrolumab in patients with moderately to severely active RA who are resistant to other biological DMARDs and who have a high type I IFN gene signature has been registered. Conversely, in patients with SS, the clinical benefits of IFN-α agonists are based on the results of early-phase trials, which suggested a potential benefit in dry mouth and salivary flow. However, larger trials, unfortunately, did not confirm these positive results [245].

### 6.3. Targeting Intracellular Signaling Pathways in Autoimmune Therapy

JAK-STAT inhibition has become a new therapeutic option for several rheumatologic conditions in recent years. JAK inhibitors (JAKi) are small oral molecules that prevent the phosphorylation of JAKs, thus interfering with the production of several inflammatory cytokines. The efficacy and safety of several JAKis, such as tofacitinib, baricitinib, upadacitinib, and filgotinib, in patients with RA, PsA, and AS have been comprehensively investigated in several clinical trials. Consequently, JAK inhibitors have been approved and registered as a treatment for patients with this inflammatory arthritis.

In patients with RA, JAKi are efficient in reducing disease activity, pain, and morning stiffness; in reaching ACR response; in suppressing radiographic joint damage progression; and in improving bone properties in patients naive to biological treatment as well as in patients with inadequate response to previous lines of therapy [246]. Thanks to these important and surprising results, JAK inhibitors now represent a possible choice for the treatment of patients with rheumatoid arthritis, according to the recent EULAR guidelines [247].

In patients with psoriatic arthritis, JAKi studies demonstrated that these drugs are effective for cutaneous and articular manifestations, for dactylitis and enthesitis, and also for slowing radiographic progression [248]. Even in this case, nowadays, they represent a therapeutic alternative in patients with psoriatic arthritis after biologic DMARD failure or in cases where biologic DMARDs are not an appropriate choice, as pointed out by the last EULAR guidelines [249]. Even in the case of axial involvement, such as patients with ankylosing spondylitis, JAKi seems to be effective, both in the reduction in clinical symptoms like pain, mobility, and morning stiffness and radiographic parameters, especially in patients with radiographic spondylitis [250]. In 2022, they were included in the ASAS-EULAR axSpA recommendations update, which advises that they can be used in patients with persistently high disease activity despite conventional treatment [251].

Since the Janus kinase-signal transducer and activator of transcription (JAK-STAT) pathway is responsible for an increase in IFN production, multiple JAK inhibitors (JAKi) are currently being considered in patients with SLE. In general, the results of phase II and phase III studies and real-life observations are not always consistent, and as a result, JAKi are not currently considered to be first-line treatments in patients with SLE, but their use is limited to clinical manifestations that do not respond to conventional treatment with traditional and biological DMARDs.

Tofacitinib, a prevalent JAK1 and JAK3 inhibitor, has been successfully tested in preclinical studies and afterwards in phase I/II clinical trials with good results in the improvement of cardiometabolic and immunologic parameters associated with the premature atherosclerosis in SLE, including type I IFN gene signature and circulating NETs [252].

The results of a retrospective analysis of a SLE Chinese cohort (CSTAR: Chinese SLE Treatment and Research Group) involving 109 patients with active mucocutaneous and joint involvement have recently been published. Patients treated with tofacitinib experienced a better resolution of these manifestations when compared to methotrexate, with a comparable safety profile [253].

Mucocutaneous manifestations such as alopecia, as well as arthritis, seem to be the kind of manifestations that respond best to the addition of JAK inhibitors such as tofacitinib, confirming that these drugs may be an effective therapeutic option for treating patients with these involvements, especially when they have been resistant to previous treatments.

Another JAK inhibitor, baricitinib, has been more extensively studied in patients with SLE, even in this case showing efficacy in controlling skin and joint manifestations [254].

Despite the positive results of the initial trials and the satisfactory results of several case reports described in the literature in patients with cutaneous, musculoskeletal, and renal involvement, the two phase 3 trials, SLE-BRAVE I and SLE-BRAVE II, failed to reach the objectives [255]. The efficacy of baricitinib in renal SLE has been tested in another clinical trial registered with CTI: NCT0543253; however, the study seems to be finished, but the results have not been submitted yet.

More recently, a newer generation JAKi, which selectively inhibits JAK1, upadacitinib, has proven to be effective in patients with moderate to severe SLE, and these positive results have also been confirmed by the long-term extension study. Upadacitinib seems to be able to reduce steroid intake, activity of disease, and type I IFN pathway protein expressions [256]. Currently, a new phase III study (CTI: NCT05843643) to assess the efficacy and safety of upadacitinib in patients with moderate to severe SLE has been launched, and the recruiting phase is ongoing.

Finally, another JAK1 inhibitor, filgotinib, has been used in a phase II multicenter study in patients with class V lupus nephropathy, demonstrating good results, despite the small number of participants [257], but there are no larger studies currently underway with this drug.

The JAK/STAT pathway also takes part in the process of SS by affecting many cytokine signals, and several basic studies suggest that JAK inhibitors may be effective for pSS.

Currently, there are several studies that suggest the efficacy of this class of drugs in Sjogren’s syndrome; however, more consistent results are needed to be able to use them more safely and consciously in our clinical practice.

One of the first drugs in this class to be tested with laboratory studies was tofacitinib. The efficacy of tofacitinib has been studied in one retrospective and one prospective cohort of patients with SS, analyzing changes in clinical and laboratory parameters, as well as modifications in circulating T cell subsets. Despite several limitations in study design, tofacitinib improves multiple parameters, suggesting its potential use as a therapeutic option in SS patients [258].

Following the positive results of the pilot study on baricitinib in SS patients in terms of reduction in activity of analyzing disease [259], this drug has also been taken into account, and a multicenter randomized study is now being performed to assess the clinical efficacy and safety in adult subjects with Sjogren’s syndrome (CTI: NCT05016297).

Filgotinib, a JAK-1 preferential inhibitor, has been tested, together with other TYK inhibitors, in a recent randomized trial. Although either primary or secondary endpoints were not met, it seems that a group of patients with highly active systemic disease, based on high-sensitivity C-reactive protein and high patient-reported SS symptoms, could benefit from the therapy with filgotinib [260].

The use of JAK-i is also increasing in systemic sclerosis; through inhibition of multiple inflammatory cytokines, JAK-I could also block critical mediators in the fibrotic process, thereby reducing skin and visceral fibrosis.

A recent systematic review on the use of JAK inhibitors in systemic sclerosis shows that there are almost a hundred cases of patients successfully treated with JAK inhibitors, and in most cases, the type of disease involvement was a combination of skin and interstitial or skin and gastrointestinal involvement.

Tofacitinib was used by most patients, and an improvement was reported in almost 90% of cases, while adverse effects were reported by approximately half of the patients [261].

JAK inhibitors could represent a promising tool in these patients, and their efficacy and safety need to be confirmed with further studies. While JAK inhibitors exhibit promising therapeutic potential for several rheumatic diseases, they have potential side effects that warrant careful consideration. JAK-STAT pathways are also involved in key biological processes like adipogenesis and hematopoiesis; therefore, these drugs could cause various predictable side effects.

Moreover, regarding safety, the ORAL Surveillance study found that patients with RA aged 50 or more who had at least one additional cardiovascular risk factor, if treated with tofacitinib, had an increased risk of cardiovascular events compared with those treated with TNFi and also an increased risk of malignancy [262].

Similarly, baricitinib and TNF inhibitors were compared in patients with RA in an observational study: baricitinib was associated with a higher incidence of venous thromboembolism and major cardiovascular events [263].

As a result, both the European Medicines Agency (EMA) and the U.S. Food and Drug Administration (FDA) have issued a statement cautioning against the use of all JAKi as the first option in patients over 65 years of age, smokers, and those with cardiovascular risk factors, a history of thromboembolic events, or a history of malignancy.

Nintedanib, a small-molecule tyrosine kinase inhibitor able to turn off growth factors such as TGF-beta, FGF and PDGF receptors, has gained significant attention for its antifibrotic properties, particularly in the management of interstitial lung disease (ILD) associated with various autoimmune and connective tissue diseases [264]. The primary indication for nintedanib in RA is in patients who develop progressive fibrosing interstitial lung disease (RA-ILD). Clinical studies, including the INBUILD trial (CTI: NCT02999178), have demonstrated that nintedanib significantly slows the decline in forced vital capacity (FVC) in these patients and in patients with other connective tissue disease-ILD (CTD-ILD). Specifically, the INBUILD trial showed a 57% reduction in the rate of FVC decline over 52 weeks compared with placebo, with a safety profile consistent with gastrointestinal adverse events, primarily diarrhea, which were generally manageable [265]. The drug’s efficacy and safety were consistent whether or not patients were on disease-modifying antirheumatic drugs (DMARDs) or glucocorticoids, and real-world evidence supports its effectiveness and tolerability in a broader clinical context, including in older populations with longer disease duration and comorbidities, although adverse events sometimes necessitate dose reduction or discontinuation [266,267]. Strong evidence supports the use of nintedanib in ILD associated with systemic sclerosis (SSc-ILD). The pivotal phase III SENSCIS study (CTI: NCT02597933) established that nintedanib significantly reduces the annual rate of decline in FVC in these patients, regardless of patients’ extent of fibrotic SSc-ILD [268]. Also, Sjögren’s syndrome can be complicated by ILD, and nintedanib has been used in cases of progressive disease where fibrosis is a prominent feature [269]. However, nintedanib is associated with certain side effects and cannot halt the progression of the disease or reverse the decline in lung function [270]. The availability of methods capable of detecting a patient’s response to the drug in order to monitor the effectiveness of treatment would be extremely valuable for clinical management. Promising results obtained have identified the serum BAG3 protein as a biomarker of SSc [271,272], and a limited number of cases have shown that it can also be useful in monitoring patients’ clinical response to nintedanib therapy [273].

## 7. Conclusions

Autoimmune diseases exhibit significant heterogeneity and complex cytokine networks, making personalized medicine essential for optimal treatment. Biomarker-driven strategies enable tailored cytokine and growth factor modulation, improving efficacy while reducing adverse effects. This approach relies on molecular, genetic, and immunologic biomarkers to stratify patients and customize therapies. Combination treatments targeting multiple cytokines or integrating cytokine inhibitors with conventional immunosuppressants are being explored to enhance synergy, control disease better, and overcome partial responses. Nevertheless, challenges remain, including variable patient responses, infection risks, and compensatory cytokine pathways that limit durability.

Advances in multiplex biomarker profiling, genomics, transcriptomics, and epigenomics, combined with machine learning, are facilitating the development of next-generation, patient-specific therapies. Integration of genetic, proteomic, and clinical data helps predict individual treatment responses and identify non-responders early. Furthermore, emerging modalities such as nanobodies, exosome-based delivery, and gene editing offer higher target specificity, improved drug stability, and reduced systemic effects, all aiming to achieve more precise and durable cytokine-targeted treatments.

In summary, the future of cytokine and growth factor modulation in autoimmune diseases is poised for a transformative leap through the fusion of biomarker-driven personalized medicine, cutting-edge therapeutic technologies, and innovative combination strategies. This combination will allow the delivery of treatments that are not only more effective but also safer and finely tailored to individual patient profiles.

As reported and discussed in this review, it is clear that cytokines and growth factors play a pivotal role as master regulators of immune function, where their dysregulation drives both the initiation and perpetuation of autoimmune pathology. Their dual capacity to mediate inflammation and immune regulation underscores the necessity of fine-tuned cytokine targeting, which only personalized approaches can achieve. By leveraging integrated biomarker information and advanced delivery systems, future therapies aim to recalibrate cytokine networks precisely, minimizing collateral immune suppression and enhancing long-term disease remission.

## Figures and Tables

**Table 1:** Overview of key cytokine functions in autoimmune disease pathogenesis.

CYTOKINES	CELL SOURCES	MAIN TARGETS	MAIN ROLES	ASSOCIATED RHEUMATIC DISEASE	THERAPEUTIC AGENTS
TNF-α	T and NK lymphocytes, macrophages	Th1 and Th17 cells, monocytes, macrophages, endothelial cells, synovial fibroblasts, osteoblasts, osteoclasts	Release of inflammatory mediators, promotion of innate and acquired immune response, fever induction, joint and bone erosion, tissue damage (eg. glandular tissue), formation of ectopic lymphoid structures	RA, SLE, SS, PsA, AS	Infliximab, etanercept, adalimumab, certolizumab, golimumab, ozoralizumab
IL-1-β	Monocytes, macrophages	T cells, dendritic cells, fibroblast-like synoviocytes chondrocytes, osteoclasts, fibroblasts	Lymphocyte activation and proliferation, synthesis of collagenases, COX-2, proteases inhibitors and MMPs that contribute to cartilage degradation, endothelial cell activation, differentiation of fibroblasts into myofibroblasts	RA, SLE, SS, PsA, SSc	Anakinra, canakinumab, rilonacept
IL-6	B and T cells, macrophage, fibroblasts, keratinocyte, endothelial cells, mast cells and dendritic cells	Monocytes, T and B cells, synoviocytes, osteoclasts, fibroblasts	Activation of acute-phase responses and immune mechanisms, regulation of body weight, support of liver function and bone health, promotion of angiogenesis and pannus formation, Th17 differentiation and activation	RA, SLE, SS, PsA, AS, SSc	Tociliizumab, sirukumab, sarilumab, siltuximab, JAK-inhibitors (tofacitinib, baricitinib, upadacitinib, filgotinib)
IL-17	Th 17 cells and others T cells	Epithelial cells, fibroblasts, osteoclasts, dendritic cells	Activation of immune responses, maintenance of barrier integrity, production of inflammatory mediators, Th17 differentiation and activation, promotion of tissue repair and fibrosis	RA, SLE, SS, PsA, AS, SSc	Secukinumab, bimekizumab, risankizumab
IFN-γ	Plasmacytoid dendritic cells, CD4+ T cells	Macrophages, dendritic cells, epithelial cells, endothelial cells, leukocytes, adipose tissue cells	Cytokines production, antigen presentation, generation of reactive oxygen species, regulation of metabolic processes, macrophage polarization toward the pro-inflammatory M1 phenotype, modulation of cell growth and survival	SLE, SS, SSc	Anifrolumab, JAK-inhibitors (tofacitinib, baricitinib, upadacitinib, filgotinib)
IL-10	B and T cells, monocytes, macrophages, dendritic cells	B and T cells, macrophages, neutrophils, myeloid cells	Suppression the excessive pro-inflammatory responses; macrophage polarization toward the M2 phenotype	RA, SS, AS	rhIL-10
IL-4	Th 2 cells, eosinophils, basophils and mast cells	B and T cells, macrophages	Regulation of B cell differentiation, survival and memory formation; influence on differentiation of T-cell subset; tissue repair; resolution of inflammation; macrophage polarization toward the M2 phenotype	SLE, SS, PsA	Dupilumab
TGF-β	Fibroblasts, macrophage, endothelial cells, platelets, T cells, epithelial cells	Epithelial cells, fibroblasts, B and T cells, macrophages	Immune tolerance promotion, development and differentiation of T-cell subset, wound healing	SSc	Nintedanib

## Data Availability

Data sharing is not applicable.

## Abbreviations

The following abbreviations are used in this manuscript:

RA	Rheumatoid Arthritis
SLE	Systemic Lupus Erythematosus
PsA	Psoriatic Arthritis
SS	Sjögren’s Syndrome
AS	Ankylosing Spondylitis
SSc	Systemic Sclerosis
